# Numerical and Experimental Investigations of Polymer Viscoelastic Materials Obtained by 3D Printing

**DOI:** 10.3390/polym13193276

**Published:** 2021-09-25

**Authors:** Jusuf Ibrulj, Ejub Dzaferovic, Murco Obucina, Manja Kitek Kuzman

**Affiliations:** 1 Mechanical Engineering Faculty, University of Sarajevo, Vilsonovo setaliste 9, 71000 Sarajevo, Bosnia and Herzegovina; dzaferovic@mef.unsa.ba (E.D.); obucina@mef.unsa.ba (M.O.); 2 Biotechnical Faculty, University of Ljubljana, Jamnikarjeva 101, 1000 Ljubljana, Slovenia; manja.kuzman@bf.uni-lj.si

**Keywords:** relaxation modulus, creep modulus, viscoelasticity, numerical, experimental, Prony series

## Abstract

The aim of this research is to determine the relaxation and creep modulus of 3D printed materials, and the numerical research is based on the finite volume method. The basic material for determining these characteristics is ABS (acrylonitrile butadiene styrene) plastic as one of the most widely used polymeric materials in 3D printing. The experimental method for determining the relaxation functions involved the use of a creep test, in which a constant increase of the stress of the material was performed over time to a certain predetermined value. In addition to this test, DMA (dynamic mechanical analysis) analysis was used. Determination of unknown parameters of relaxation functions in analytical form was performed on the basis of the expression for the storage modulus in the frequency domain. The influence of temperature on the values of the relaxation modulus is considered through the determination of the shift factor. Shift factor is determined on the basis of a series of tests of the relaxation function at different constant temperatures. The shift factor is presented in the form of the WLF (Williams-Landel-Ferry) equation. After obtaining such experimentally determined viscoelastic characteristics with analytical expressions for relaxation modulus and shift factors, numerical analysis can be performed. For this numerical analysis, a mathematical model with an incremental approach was used, as developed in earlier works although with a certain modification. In the experimental analysis, the analytical expression for relaxation modulus in the form of the Prony series is used, and since it is the sum of exponential functions, this enables the derivation of a recursive algorithm for stress calculation. Numerical analysis was performed on several test cases and the results were compared with the results of the experiment and available analytical solutions. A good agreement was obtained between the results of the numerical simulation and the results of the experiment and analytical solutions.

## 1. Introduction

Most polymeric materials are used today for their good mechanical properties in relation to manufacturing costs. For this reason, the mechanical properties of these materials are of interest because of their wide application. It is important to know the dependence of deformation and stress when analyzing the stability of elements or products exposed to a load, and it is known that the characteristics of polymeric materials change over time. A complete description of the mechanical properties of polymeric materials usually requires knowledge of the behavior of the material over 10 to 20 years [1]. This means that in order to determine the reliability and durability of a certain polymer structure, it is necessary to know the mechanical characteristics during the entire time of use.

In the theory of linear viscoelasticity, such characteristics are most often the relaxation modulus E(t) and the creep function J(t), which define the response of the material to the applied load (i.e., the Heaviside function). Moreover, due to the short duration of the experiment, it is often necessary to replace the step load function with other non-standard loads, such as a ramp function or a function that has a constant stress or strain ratio [2]. In practice, polymeric materials are often exposed to a load that has a constant stress or strain ratio over time. Uniaxial tensile loading with a linear increase in deformation over time is the most important type of load in polymer technology. It is widely used in the production of fibers, films, and thin polymer plates, as well as in the combination of these and other technological processes. However, these types of experiments are not widely used due to the complicated preparation steps and the equipment needed to provide the necessary control of the stress/strain ratio. Dynamic-mechanical analysis (DMA-tests) can also be used to efficiently determine the characteristics of polymers. These tests apply sinusoidal stress and measure the deformation of the material, allowing the determination of the storage modulus as well as the loss modulus. The stress frequency changes periodically, leading to variations in the complex modulus. If, in addition to the stress, the temperature also changes during the test, then this approach can be used to determine the glass transition temperature of the material, T_g_. 

Fused deposition modeling (FDM) technology is increasingly being applied, and as a new technology requires specialized 3D printers that use thermoplastics (polymers) as a raw material for the production of strong, durable, and dimensionally stable elements. An additional advantage of FDM technology is that the parts can have a mesh filling, according to the design requirements, which can produce very light parts that can better respond to the function. This technology has the key advantages over all other 3D element manufacturing technologies of precision and repeatability. Other advantages of this technology are its convenience, cleanliness, and ease of use, and the fact that the polymers from which the elements are made are mechanically and environmentally friendly, and therefore enable working with complex geometries that are more difficult or impossible with other technologies (e.g., casting).

The application of constitutive equations for thermoviscoelastic polymers in the finite element method has been investigated in recent years. Initially, researchers proposed a finite element formulation for rubber under low oscillatory loads. They later investigated the same material by developing a phenomenological viscoelastic constitutive model with finite loading within irreversible thermodynamic processes with changing variables. The proposed procedure is implemented in the user-defined subroutine UMAT in the software used for nonlinear finite element analysis ABAQUS/Standard, and is applicable to compressible polymers and elastomers [3,4]. 

The original 2D approach of Fryer [5,6] was subsequently extended to three dimensions by Bailey and Cross [7,8]. In addition, the finite volume method has been applied to a number of fluid-solid interaction problems, and includes the assumption of viscoelasticity of one or more materials [9,10,11,12,13,14,15,16,17,18]. These approaches are implemented in licensed as well as “open source” software (openFOAM) that allows discretization of time, space, and equations based on the finite volume method. A group of authors [19,20,21] successfully applied the finite volume method in the analysis of mechanical and rheological properties of wood, where the influence of moisture as well as that of the gluing technology used with the laminated wooden elements on the viscoelastic properties were analyzed. The same authors also considered multiparameter models and their influence on the determination of the viscoelastic properties of materials [21,22]. In the analysis of polymer 3D printed objects, the creep phenomenon was considered, using a three-parameter model, while the results obtained using the finite volume method were confirmed based on the results obtained experimentally [23].

A general overview of time-dependent characteristics of polymeric materials was given by Tschoegl [24]. Additionally, Zhao and Knaus [25] explained the physical response of linearly viscoelastic materials in detail based on ramp tests. Moreover, several methods have been proposed for the conversion of a relaxation module from a frequency to a time domain. The Prony series is very popular for the representation of viscoelastic functions because it can cover a wide time range as well as because of the computational efficiency that results from its exponential base. The collocation method for converting relaxation modules from the frequency domain to time domain was proposed by Schapery [3], while Emri and Tschoegl [24] developed a recursive algorithm that generates a line spectrum from experimental data. They applied the window method to ensure positive values of all elements in the Prony series. Ramkumar used the regularization method with quadratic programming to minimize oscillations in the experimental data. Later, Schapery and Park developed a new method of interconversion based on a numerical and approximate-analytical approach. To solve the problem of determining the coefficients in the Prony series, many tools are available in the literature, and some of these have already been implemented in commercial software packages such as MATLAB, Mathcad, Maple, Origin, etc. [1,2,3]. In this paper, the MATLAB software package with built-in subroutines for optimization was used.

One of the most important methods for determining the long-term behavior of polymers is based on the principle of temperature-time superposition (TTSP), which uses information from short-term tests at different temperatures. The method uses the similarity of the variations of the relaxation/creep modulus over time at different temperatures. This approach allows the creation of a master curve that moves based on the value of the shift factor. Later, the experimental time frame was extended to successfully show the relationship between time, creep/relaxation function values, and glass region temperature. One-dimensional tensile experiments were performed using a Meissner-type rheometer to measure ABS properties and a material model was proposed that takes into account the effects of strain rate, solidification strain, and temperature sensitivity. An innovative thermoforming process has recently been developed to form composite layers using pressure while melting the polymer during the injection phase. This process is an integration of thermoforming and injection processes, and can result in a significant reduction in the tools, machines, and operating costs needed. A very significant study of the mechanical properties of 3D printed polymeric materials was conducted by a group of researchers [25], whose subject was initially wood as a natural polymer, to extend the research to other synthetic materials (PLA, ABS, PE) [26,27,28] as well as their combinations.

Based on the results of previous research, it is possible to introduce an analytical expression in the form of a Prony series to express the values of the relaxation function during an arbitrary period of time. The unknown parameters in the Prony series can be determined by regression analysis, and the influence of temperature can be represented using the temperature-time principle of superposition. Analytical expressions derived in this way can be used in numerical analysis, and the obtained results are confirmed on the basis of conducted experiments or available analytical solution. The model developed by Demirdzic, Dzaferovic and Ivankovic [13] for the analysis of thermoviscoelastic deformations in the finite volume method is the basis for calculations, while a recursive algorithm obtained on the basis of using Prony’s series is an upgrade that enables more efficient and faster calculation of parameters.

## 2. Materials and Methods

### 2.1. Experimental Analysis

This chapter describes the experimental analysis of viscoelastic materials obtained by 3D printing. The material characteristics and basic tests for determining relaxation functions are described, as well as the tests used in this paper.

Tests with a constant increase in load over time can be considered a limit case when the time of the ramp function coincides with the total load time. Here, a constant rate of change of load over time is used instead of a constant load. Tests with a constant increase in load are typical for testing soft materials, such as gels or liquids. The most interesting and practical uses are optimization methods where relaxation functions are obtained from the process of approximating the stress or deformation of the material. Relaxation functions are represented as a sum of exponential functions, i.e., a Prony series [24]. The appropriate mathematical expression for the Prony series is:

(1) $$E\left(t\right)=E_{\infty }+\sum_{k=1}^{K}E_{k}\cdot \exp\left(-\frac{t}{\tau_{k}}\right),$$

where E_∞_, E_k_, τ_k_ are the “rubbery” relaxation modulus, coefficients in the Prony series, and relaxation times, respectively. While the appropriate formulation for the creep modulus is:

(2) $$J\left(t\right)=J_{o}+\sum_{k=1}^{K}J_{k}\left[1-\exp\left(-t/\tau_{k}\right)\right]\;,$$

where J_o_, J_k_, and τ_k_ are the coefficients in the Prony series and relaxation times in case of creep. On the other hand, the constitutive relation for a linear viscoelastic material is given in the following form:

(3) $$\sigma \left(t\right)=\int_{0+}^{t}E\left(t-\tau \right)\frac{\partial \varepsilon }{\partial \tau }d\tau \;.$$

Based on Equation (1), the following expressions can be obtained for the relaxation modulus:

(4) $$\sigma \left(t\right)=c\cdot t+\sum_{k=1}^{K}d_{k}\cdot \left[1-\exp\left(-\frac{t}{\tau_{k}}\right)\right],$$

or the creep compliance

(5) $$\varepsilon \left(t\right)=g\cdot t+\sum_{k=1}^{K}h_{k}\cdot \left[1-\exp\left(-\frac{t}{\tau_{k}}\right)\right]\;.$$

Unknown coefficients $c=E_{o}\cdot \;{\dot{\varepsilon }}_{o},{\;d}_{k}=E_{k}\cdot \;{\dot{\varepsilon }}_{o},g=J_{o}\cdot \;{\dot{\sigma }}_{o},{\;h}_{k}=J_{k}\cdot \;{\dot{\sigma }}_{o}$ are determined from the approximation or nonlinear regression procedure described in the next section.

In cases when we have the experimentally determined value of the relaxation module in tabular and/or graphical form, the task is to determine the analytical form of the relaxation module E_rel_(t). The most efficient and common method for approximating this type of result is the least squares method or its variation. This problem boils down to determining the parameters E_k_ and τ_k_ in Equations (4) and (5). If the difference of the squares between the measured values and the calculated values is expressed in the following form:

(6) $$F\left(E_{k},\tau_{k}\right)=\sum_{k=1}^{K}\sum_{l=1}^{L}{\left(E\left(t_{l}\right)-E_{k}\left(t\right)\right)}^{2}\;,$$

where L is the number of measurements. Then, from the condition that the stated sum is minimum, we have a system of nonlinear equations:

(7) $$\overset{K}{\underset{k=1}{\sum }}\frac{\partial F}{\partial E_{k}}=0,\overset{K}{\underset{k=1}{\sum }}\frac{\partial F}{\partial \tau_{k}}=0.\;$$

From the last expression (7) arises a system of nonlinear equations with as many unknowns as we have parameters in the Prony series (E_k_, i τ_k_) whose solution gives an analytical expression for the relaxation module that can be further used in numerical calculations. Numerous nonlinear optimization routines are already available in the MATLAB Optimization Toolbox. In this paper, the “lsqcurvefit” MATLAB functions were used to solve the problem of nonlinear approximation (fitting), which in turn use the least squares method or its variation with an interface designed specifically for data approximation problems.

Relaxation and creep tests are suitable for the long-term study of material responses (minutes or days), but less accurate in cases of shorter times (seconds or less). Dynamic tests are used when the stress (or strain) resulting from sinusoidal stress is often good for filling a “short” range of polymer reactions. When the viscoelastic material is subjected to a sinusoidally variable stress, the response is also sinusoidal and has the same angular frequency, but is late in the phase with an angle δ. The deformation lags behind the stress at the phase angle δ, and this also applies if the deformation is a controlled variable (input load) instead of the stress. Using Fourier transforms, the real part of the relaxation module can be expressed as follows [1,2]:

(8) $$E^{\prime }\left(\omega \right)=E_{o}+\sum_{k=1}^{K}\frac{\omega^{2}\tau_{k}^{2}E_{k}}{\omega^{2}\tau_{k}^{2}+1}\;.$$

Then, the imaginary part is:

(9) $$E^{\prime \prime }\;\left(\omega \right)=\sum_{k=1}^{K}\frac{{\omega \tau }_{k}E_{k}}{\omega^{2}\tau_{k}^{2}+1}\;,$$

where is k is the number of members in the Prony series.

The relaxation modulus of polymeric materials usually has three regions, which are determined by time and temperature.

**Figure 1:**
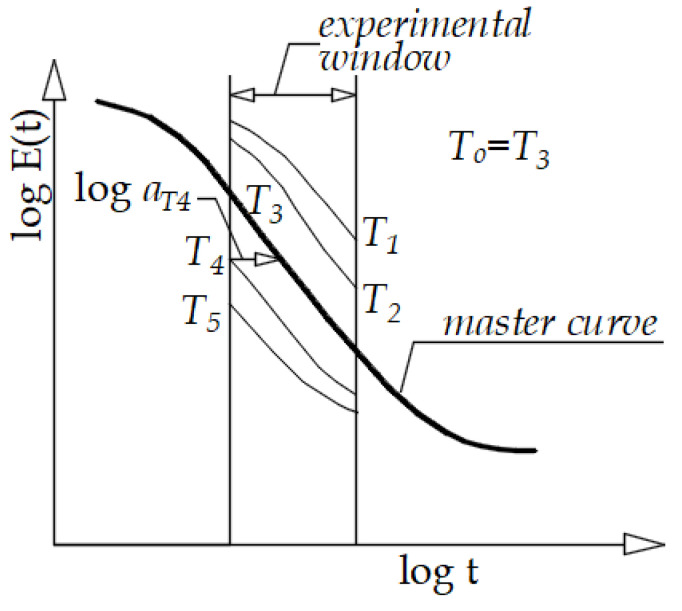
Schematic representation of a relaxation module for a polymer [24].

The first area is called glassy, where the properties are constant and the material is hard and brittle. The test material then enters a transition area where the material properties change drastically. By further increasing the temperature we reach the rubber region, where the material behaves like rubber. The relaxation modulus curve usually extends over several decades, which is physically impossible to measure. For this reason, data from experiments are collected within a certain time interval, which is usually called an experimental window (Figure 1).

In this paper, a simple CFS methodology of displacement in closed form is used, which completely eliminates the previously mentioned problems related to the displacement of experimental data in the process of construction of the main curve at the selected reference temperature [3]. Suppose that the experiments were performed at M different temperatures, or {T_j_; j = 1, 2, …, M}. Furthermore, let each of the M measured segments be given with N_j_ discrete data, then it could be written in the following relation:

(10) $$E\left(T_{j},t\right)=\left\{E_{j,i}=E_{i}\left(T_{j}\right),t_{i},\;i=1,\;2,\;3,\;\ldots .,N_{j}\;\right\}.\;$$

Usually, the experimental data of the relaxation modulus and the creep function are presented as discrete data pairs in a double logarithmic scale. Taking this into account, then the whole range of experimental data can be expressed as:

(11) $$E\left(T,t\right)=\left\{\begin{array}{c} \log E_{j,i}=\log E_{i}\left(T_{j}\right),\log t_{j,i}=\log t_{i}\left(T_{j}\right);\;\\ \;j=1,\;2\;,\;3,\ldots ,M;\;i=1,\;2,\;3,\;\ldots .,N_{j}\; \end{array}\right\}.\;$$

If we select the temperature T_κ_ as the reference temperature, then for the corresponding data measured at this temperature as the reference segment of the main curve, the following holds:

(12) $$E\left(T_{\kappa },t\right)=\log E_{\kappa ,i},{\;\log\;t}_{\kappa ,i}\;i=1,\;2,\;3,\;\ldots .,N_{\kappa }\;.\;$$

**Figure 2:**
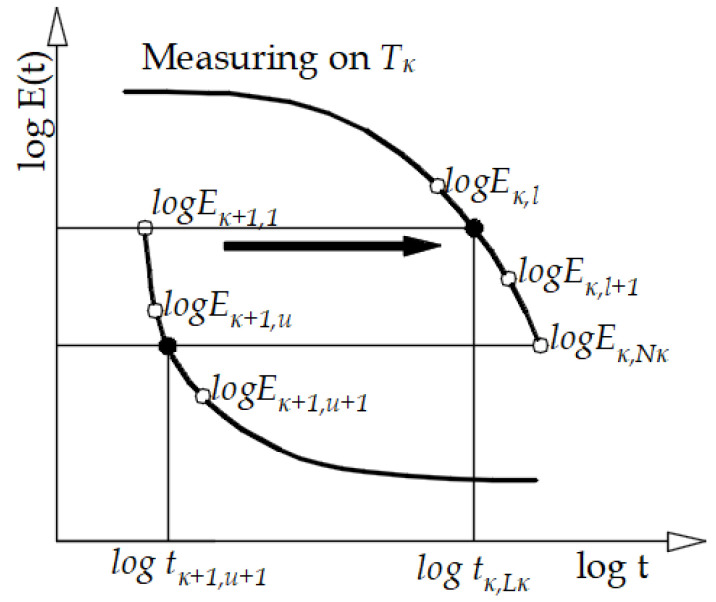
Schematic representation of the shifting procedure [2].

To construct a master curve at the selected temperature Tκ, it is necessary to move all segments measured at T_j_ > T_κ_ to the right, and the remaining segments, measured at T_j_ < T_κ_ to the left along the logarithmic time axis, so as to obtain a “smooth” main curve, as shown in the schematic in Figure 2. The individual horizontal displacements, log a (T_j_, j ≠ κ) obtained in this process, represent a set of discrete values describing the so-called time-temperature superposition, which is usually modeled with the WLF expression.

(13) $$\log a_{T}=\frac{-C_{1}\left(T_{i}-T_{o}\right)}{C_{2}+T_{i}-T_{o}}\;.$$

C_1_, C_2_–WLF constants and are determined experimentally, in which T_o_ is the reference temperature for which the master curve is constructed, and T_i_ is the current temperature.

The research samples were printed from Z-ABS ABS filament (Zortrax, Poland) with a Zortrax M200 3D printer. A 0.4 mm nozzle and a 1.75 mm diameter thread were used to print 50 × 12 × 4 mm samples for rheometer tests and 80 × 10 × 4 mm for bending tests. The print nozzle temperature was 275 °C and the printer bay temperature was 80 °C. Samples with different layer thicknesses of 0.09 mm, 0.19 mm, and 0.39 mm were printed. The samples had the same structure—the three outer layers were filled with material, and the inner structure was a mesh structure with a square size of 1.25 mm and a line thickness of 0.4 mm.

**Figure 3:**
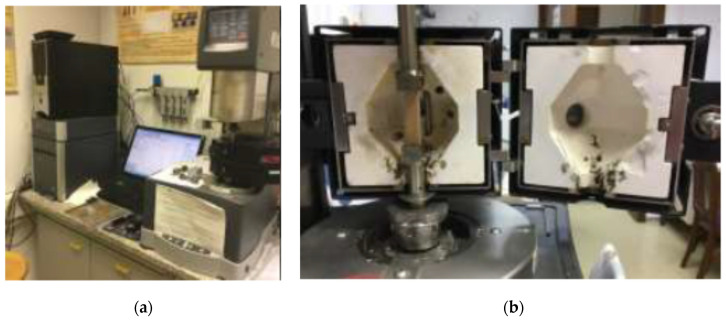
Rheometer ARES-G2 (**a**) and sample placed in torsion clamp and in temperature-controlled chamber (**b**).

**Figure 4:**
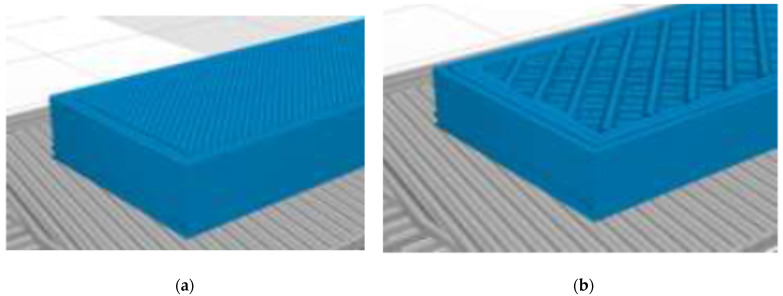
Samples with “printing” of material in three outer layers (**a**), mesh inner structure (**b**).

The rheometer (Figure 3) “ARES-G2 TA instruments” was used in the research for dynamic mechanical analysis of samples. The specimens (Figure 4) were attached to the torsional rectangular fixture and the torsional sinusoidal load. The test procedure was controlled by computer Trios software. The amplitude test with oscillating amplitude was performed at 1 Hz with a stress increase of 1.0 × 10_−6_ to the end of the linear viscoelastic region of the material, which was determined by a sudden change (more than 5%) of the storage modulus. The tests were performed at a constant temperature of 30 °C. The storage modulus, loss modulus, and tan δ are calculated from the material response measurements.

Oscillatory rheometry is one of the DMA-like techniques used to investigate the mechanical properties of the materials. The material is oscillating, and the response is measured by different methods. The response can be elastic (represented by elastic or storage modulus (G rolled cous (represented by modulus of loss (G″). For real materials the response is a combination of elastic and viscous response. The value tan (δ) is the ratio between modulus of loss and storage module and is sometimes called the dumping factor. Tensile tests of the samples were performed on a universal testing machine Zwick-Roell Z005 with a constant load speed of 1 mm/min.

### 2.2. Numerical Method

This chapter presents a mathematical model to describe the deformations of a viscoelastic solid, including the equation of momentum, the equation of thermal energy, the constitutive relations as well as the initial and boundary conditions for the solution domain [13].

#### 2.2.1. Governing Equations

The law of conservation of momentum and the law of conservation of energy of a viscoelastic solid, of volume V located inside a closed surface S, can be expressed mathematically in the following form [13]:

(14) $$\frac{\partial }{\partial t}\int_{V}\rho \left(\frac{\partial u}{\partial t}\right)\mathrm{dV}=\int_{S}\delta \sigma \cdot n\cdot \mathrm{dS}+\int_{V}\rho \cdot {\delta f}_{b}\cdot \mathrm{dV}\;,$$

(15) $$\frac{\partial }{\partial t}\int_{V}\rho \cdot e\cdot \mathrm{dV}=-\int_{S}q\cdot n\cdot \mathrm{dS}+\int_{V}\rho \cdot s_{e}\cdot \mathrm{dV}\;,$$

where t is the time, ρ is the density, u is the displacement, δσ is the stress increment, n is the outer normal of surface S, δf_b_ is the increment of force, e is the internal energy, q is the heat flux, and $s_{e}\;$the source term.

#### 2.2.2. Constitutive Relations

For an isotropic viscoelastic material, the stress history including thermal effects is described by a hereditary integral [13]:

(16) $$\begin{array}{c} \sigma =\int_{0}^{t}2\mu \left(t-\tau \right)\frac{\delta \varepsilon \left(\tau \right)}{\delta \tau }d\tau +\\ I\int_{0}^{t}\left[\lambda \left(t-\tau \right)\mathrm{tr}\frac{\delta \varepsilon \left(\tau \right)}{\delta \tau }-3K\left(t-\tau \right)\alpha \left(T\right)\frac{\delta T\left(\tau \right)}{\delta \tau }\right]d\tau \; \end{array}\}\;,$$

where μ(t − τ) i λ(t − τ) are the Lame relaxation functions, δε(t) is the strain tensor increment, τ is the incremental time, K(t − τ) is the volume relaxation modulus, α(T) is the thermal expansion, I is the unit tensor, and T is the body temperature. It is assumed that the relations of the Lame relaxation functions and parameters for elastic materials also apply to viscoelastic materials, and that they are defined as follows:

(17) $$\begin{array}{c} \mu \left(t\right)=\frac{E\left(t\right)}{2\left[1+\upsilon \left(t\right)\right]};\\ \lambda \left(t\right)=\frac{\upsilon \left(t\right)E\left(t\right)}{2\left[1+\upsilon \left(t\right)\right]\left[1-2\upsilon \left(t\right)\right]};\\ K\left(t\right)=\frac{E\left(t\right)}{3\left[1-2\upsilon \left(t\right)\right]} \end{array}\}\;,$$

where E(t) is the Young’s relaxation modulus, and ν(t) is the Poisson number.

In the case of non-isothermal changes in the viscoelastic material, then times t and τ are replaced by the reduced times using the following next equation:

(18) $$t^{\prime }=\int_{0}^{t}\frac{\mathrm{dt}}{a_{T}\left(T\right)};\;.$$

The time temperature shift factor a_T_(T) is the material characteristic that gives the relationship between the properties of the material at temperature T and the reference temperature T_0_. The relation between the heat flux and the temperature gradient is given by Fourier’s law:

(19) q=−k·gradT (19),

where k is the thermal conductivity. The relationship between thermal energy and temperature is given by the following expression:

(20) e=c·T ,

where c is the specific heat.

#### 2.2.3. Initial and Boundary Conditions

The equation for a solid is of the hyperbolic type, and at time t = t_o_ the displacement increment and the time gradient of the displacement increment at all points of the solution domain are given as follows [13]:

(21) $$du\left(r,{\;t}_{o}\right)=du^{o}\left(r\right),\;\frac{d\left(du\left(r,{\;t}_{o}\right)\right)}{\mathrm{dt}}=d{\dot{u}}^{o}\left(r\right),\;$$

The boundary conditions must be specified on all boundary areas of the resolution domain. They can be of the Dirichlet and/or Von-Neumann type depending on whether the dependent variables or gradient values of the dependent variables are given at the domain boundary.

Dirichlet boundary conditions:

(22) $$\varphi \left(r,{\;t}_{o}\right)=f_{1}\left(t\right),$$

where φ is T, d**u**.

Neumann boundary conditions:

(23) $$\mathrm{grad}\varphi \left(r,{\;t}_{o}\right)=f_{2}\left(t\right).$$

In many practical cases, the problem can be solved only in one part of the solution domain, because there is a symmetry of the solution in the remaining area on the axis of symmetry.

#### 2.2.4. Correction of Boundary Conditions

**Figure 5:**
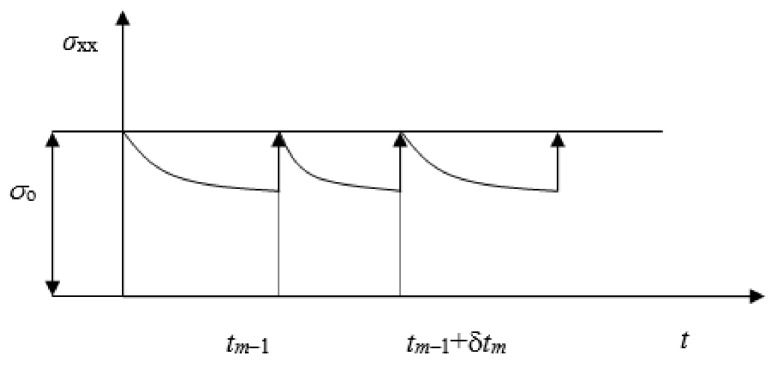
Correction of boundary condition [15].

For the case when the increment of force is given as a boundary condition at the boundary of the solution domain, due to the relaxation of the total stress, which follows from the form of the constitutive relation in which relaxation functions are used which are monotonically decreasing, it is necessary to correct the boundary condition as follows [13]:

Figure 5 shows the stress relaxation in the case of uniaxial load, when a constant stress σ_o_ is given at the limit. If f_2_(t_m_) is the total force at time tm, and df_2_(t_m_) is the increment of the force in the m−th time and/or incremental step (Figure 5), then, using the expression for total stress [15], it holds that:

(24) $${\mathrm{df}}_{2}\left(t_{m}\right)=f_{2}\left(t_{m}\right)-f_{2}\left(t_{m-1}\right)+{\mathrm{df}}^{r}\left(t_{m}\right)\;,$$

where:

(25) $${\mathrm{df}}^{r}\left(t_{m}\right)=f_{2}\left(t_{m-1}\right)-\sigma \left(t_{m-1}\right)\cdot s_{B}\;,$$

the residual increment of force,${\;s}_{B}\;$is the boundary surface vector at the domain boundary. Stress σ(t_m_) is total stress at time t_m−1_ which is relaxed at time t_m_.

#### 2.2.5. Calculation of Total Stress

The values of stress tensor σ(t_m_) are determined in the centers of the control volumes adjacent to the boundary surfaces of the solution domain. The calculation of the total stress can be done using the constitutive relation (16). By introducing the relaxation module from expression (1) into the constitutive relation, we have the following equation:

(26) $$\sigma \left(t\right)=\int_{0+}^{t}E_{\infty }\frac{\partial \varepsilon }{\partial \tau }d\tau +\sum_{k=1}^{K}\int_{0+}^{t}E_{k}\cdot \exp\left(-\frac{t-\tau }{\tau_{k}}\right)\frac{\partial \varepsilon \left(\tau \right)}{\partial \tau }d\tau \;.$$

The first term on the right side of the equation can be considered elastic while the second term is viscoelastic and can be further separated:

(27) $$\begin{array}{c} \sigma \left(t\right)=E_{\infty }\varepsilon \left(t\right)+\sum_{k=1}^{K}\int_{0+}^{t_{m}}E_{k}\cdot \exp\left(-\frac{t_{m-1}+∆t-\tau }{\tau_{k}}\right)\frac{\partial \varepsilon \left(\tau \right)}{\partial \tau }d\tau =\\ E_{\infty }\varepsilon \left(t\right)+\overset{K}{\underset{k=1}{\sum }}\exp\left(-\frac{∆t}{\tau_{k}}\right)\cdot \int_{0+}^{t_{m-1}}E_{k}\cdot \exp\left(-\frac{t_{m-1}-\tau }{\tau_{k}}\right)\cdot \frac{\partial \varepsilon \left(\tau \right)}{\partial \tau }d\tau +\\ \sum_{k=1}^{K}\int_{t_{m-1}}^{t_{m}}E_{k}\cdot \exp\left(-\frac{t_{m}-\tau }{\tau_{k}}\right)\frac{\partial \varepsilon \left(\tau \right)}{\partial \tau }d\tau \end{array}\}$$

Individual terms in Equation (27) can be written as: $\sigma_{\mathrm{elas}}=E_{\infty }\left[\varepsilon \left(t_{m}\right)-\varepsilon \left(t_{0}\right)\;\right]$ is the elastic part of the total stress. $\sigma_{\mathrm{visco}}^{m-1}=\sum_{k=1}^{K}\exp\left(-\frac{∆t}{\tau_{k}}\right)\int_{0+}^{t_{m-1}}E_{k}\cdot \exp\left(-\frac{t-\tau }{\tau_{k}}\right)\frac{\partial \varepsilon }{\partial \tau }d\tau \;$is the viscoelastic part of the total stress from the previous time step t_m−1_. The remaining members are calculated according to the following relation:

(28) $$\begin{array}{c} \sigma_{\mathrm{visco}}^{\mathrm{incr}}=\sum_{k=1}^{K}\int_{t_{m-1}}^{t_{m}}E_{k}\cdot \exp\left(-\frac{t-\tau }{\tau_{k}}\right)\frac{\partial \varepsilon \left(\tau \right)}{\partial \tau }d\xi =\\ \sum_{k=1}^{K}\frac{\varepsilon \left(t_{m}\right)-\varepsilon \left(t_{m-1}\right)}{\frac{∆t_{m}}{\tau_{k}}}\;\left[1-\exp\left(-\frac{\Delta t}{\tau_{k}}\right)\right]\; \end{array}\}\;.$$

The formula for calculating the total stress can be written as follows:

(29) $$\sigma \left(t\right)=\sigma_{\mathrm{elas}}+\sigma_{\mathrm{visco}}^{m-1}+\sigma_{\mathrm{visco}}^{\mathrm{incr}}\;.$$

It can be seen from relation (29) that to calculate the value of the total stress at the current moment t_m_ it is sufficient to know the values of the viscoelastic part of the stress from the previous time step ($\sigma_{\mathrm{visco}}^{m-1}$) as well as the deformation values from these two time steps.

## 3. Results

### 3.1. Experimental Results

This chapter presents the results of processing experiments in which a number of tests were applied, such as the test with constant increase in load (stress/strain), creep test, dynamic frequency test, dynamic amplitude test, and test for determining the temperature shift factor. All these tests were performed on the previously described experimental line and devices that give sufficient reading accuracy for the results to be considered reliable. Based on the performed experiments, the expressions for creep modulus, relaxation modulus and temperature shift factor were determined. After such analytical expressions in the mathematical form of the Prony series with knowledge of other physical characteristics of the material, numerical simulations were performed.

#### 3.1.1. Determination of Creep Modulus from Creep Test

**Figure 6:**
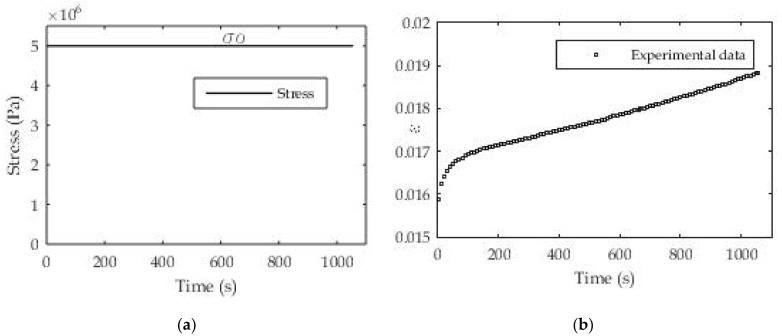
(**a**) Creep test with constant load (σ_o_ = 5 MPa); (**b**) experimental results for strain ε(t).

The well-known Heaviside function shown in Figure 6a is used to determine the creep modulus in this example. As a widely used function for a mechanical test, there are no particular difficulties in controlling the input, in this case the stress (force) since it is a creep test. The result is a deformation (creep function) during the test time ε(t) (Figure 6b).

For these tests, we also chose stress values so that the total deformation does not exceed 2% because a pronounced creep characteristic is expected in this area. In order to demonstrate the usefulness and applicability of the proposed procedure to different polymeric printed materials, we use real experimental data measured at constant load (σ_o_ = const) for the creep test. To determine the relaxation function in this case, which is the creep modulus, Equation (5) is used, i.e., the unknown coefficients J_i_ and τ_i_ are determined. The nonlinear regression procedure is implemented in MATLAB software and applied here.

**Figure 7:**
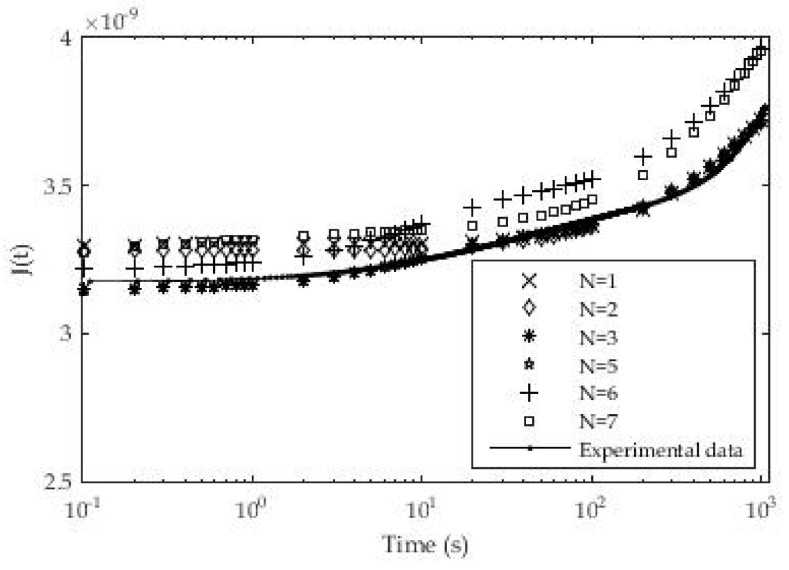
Experimental and approximate values of creep modulus (PLA, d_1_ = 0.09 mm).

Material samples were prepared on the described experimental line [23] or 3D printer in a layer thickness of value d_1_ = 0.09 mm. The dimensions of the samples used in these test cases are 50 × 12 × 4 mm. The results of testing and approximation are presented in Table 1, while in Figure 7 a graphical presentation of the results of approximation and experimental results is given. The number of exponential terms in the series was systematically increased from *n* = 1 for the roughest approximation to *n* = 7 for the finest. From Table 1 it can be seen that the optimization was performed when choosing the relaxation times, which means that the relaxation times are assumed to be known in order to reduce the number of unknown parameters and speed up the procedure for solving systems of nonlinear equations.

The coefficients in Table 1 were applied in the expression for the creep modulus and compared with the experimentally determined values. Figure 7 for the PLA material and layer thickness d = 0.09 mm shows different deviations of the fitted parameter values from the experimental ones for different numbers of exponential members in the creep module. In the initial phase of the test, a larger deviation of the fitted ones from the experimental values was noticed for the number of members *n* = 1 and *n* = 2, while for *n* = 6 and *n* = 7 a larger deviation was noted at the end of the test. The best results are obtained for the number of exponential terms *n* = 3 where the maximum deviation of experimental and fitted results is up to 1.85%. The maximum deviation for *n* = 1 at the beginning of testing is 2.9% while for *n* = 7 it is 5.3%.

In Table 2 the values of the average deviation between the fitted and experimental data for the whole test period are shown. The results show that the best solution is the Prony series with *n* = 7 exponential terms.

#### 3.1.2. Determination of Creep Modulus from Constant Stress-Rate Test

**Figure 8:**
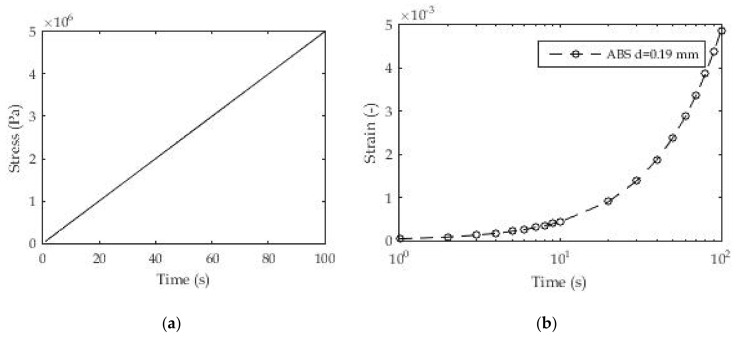
(**a**) Constant stress-rate test; (**b**) experimental results for strain ε(t).

Mechanical tests with a linear increase in load (stress/strain) over time are often used to determine creep modulus (Figure 8). This is a very demanding test and is reflected primarily in load control over time. However, this problem can be overcome quite well with the advent of the newer generation of rheometers.

For this test, we selected the same 3D printed polymeric material (PLA) with samples printed in three-layer thicknesses. The aim of this test is to determine the creep modulus or more precisely to determine the coefficients in the Prony series for the creep modulus during the test time. For this test, the number of exponential terms in the Prony series was taken from *n* = 1, 2, 5, and 10 with the relaxation times based on previous experiments and the linear distributions over several decades. The assumption of known relaxation times is based on previous similar research, and the approach used in this study is more convenient due to the reduction in the number of unknowns and equations in the system of nonlinear equations, as a consequence of the application of nonlinear regression.

Figure 8 shows the method of loading 3D printed samples of polymeric material and the linear stress ratio over time. The dimensions and geometry of the samples are identical to those in Section 3.1.1. Figure 8b shows the experimental results for deformation over time as a consequence of the applied load for one layer (d = 0.19 mm) of the 3D printed samples. All results in the form of certain coefficients were obtained using the appropriate Equation (5) in relation to the experimental results.

**Figure 9:**
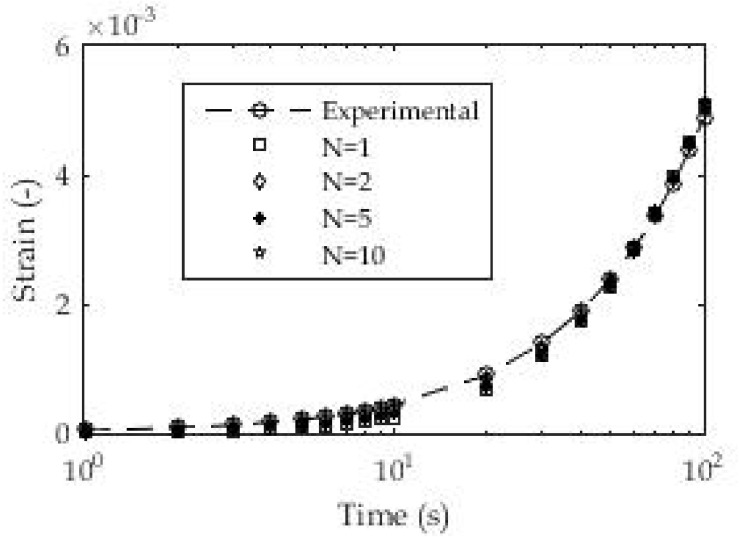
Experimental and fitted values of strain (ABS, d = 0.19 mm).

These coefficients are shown in Table 3. Figure 9 shows the good agreement of the results layer with regard to the d = 0.19 mm layer of 3D printed ABS. It is also noticeable that the results match throughout the test time, unlike the previous classic creep test with a constant stress value. In these tests, the logarithmic distribution of relaxation times in individual terms of the Prony series was used, which gave quite good fitting results even for a small number of exponential terms. Unlike the previous test, a certain consistency is shown here in terms of reducing the mean deviation of the fitted from the experimental results with an increase in the number of exponential terms. All these deviations are less than 2%, which can be assessed as quite acceptable.

#### 3.1.3. Determination of Relaxation Modulus from the Constant Strain-Rate Test

For this test, we selected identical 3D printed polymeric material (ABS) from the previous Section 3.1.2 with samples printed in layer thickness d = 0.19. The aim of this test is to determine the relaxation modulus or more precisely to determine the coefficients in the Prony series for the relaxation modulus (E(t)) during the test time.

**Figure 10:**
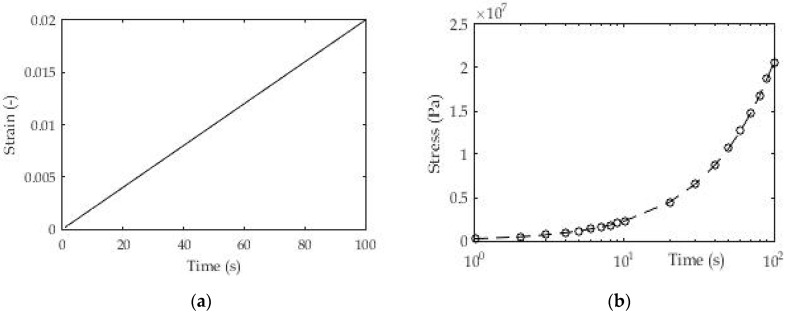
(**a**) Constant strain-rate test; (**b**) Experimental results for stress σ(t).

As in the previous test, the number of exponential members in the Prony series was taken from *n* = 1, 2, 5, and 10 (Table 4), but this time the relaxation times were taken from previous research. Figure 10 shows the method of loading 3D printed samples of polymeric material and shows the linear stress ratio over time. The dimensions and geometry of the samples are identical to those in Section 3.1.1. and Section 3.1.2.

**Figure 11:**
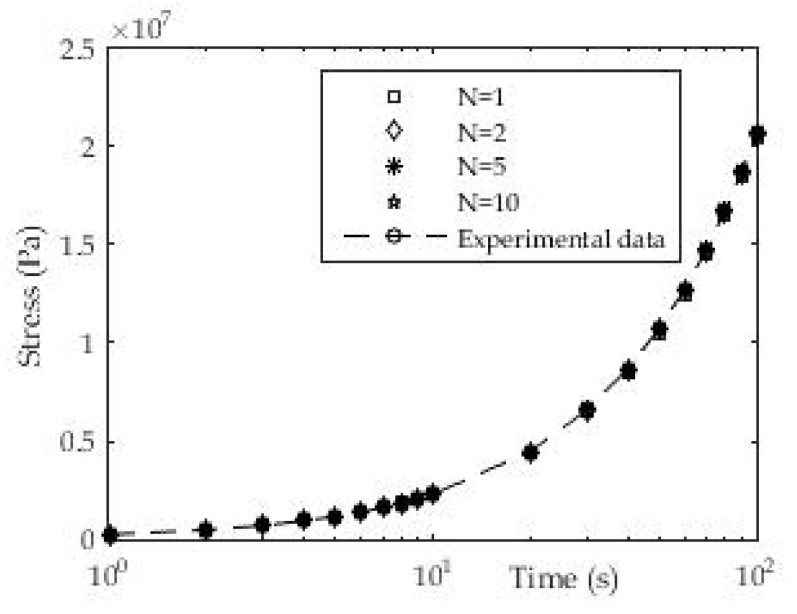
Experimental and fitted values of stress (ABS, d = 0.19 mm).

Figure 11 shows the experimental results for stress over time as a consequence of the applied load for layer thickness d = 0.19 mm of 3D printed samples. All results in the form of certain coefficients were obtained using the appropriate expression (5) for stress (σ(t)) in relation to the experimental results.

Similar to the test case of a constant increase in stress, the use of this test allowed a fairly precise determination of the relaxation function even with a small number of members in the Prony series. The previously adopted logarithmic distribution of relaxation times and the use of the derived expression (5) gave deviation values of fitted and experimental results that are less than 2%.

#### 3.1.4. Determination of Relaxation Module from Dynamic Test “Frequency Sweep”

**Figure 12:**
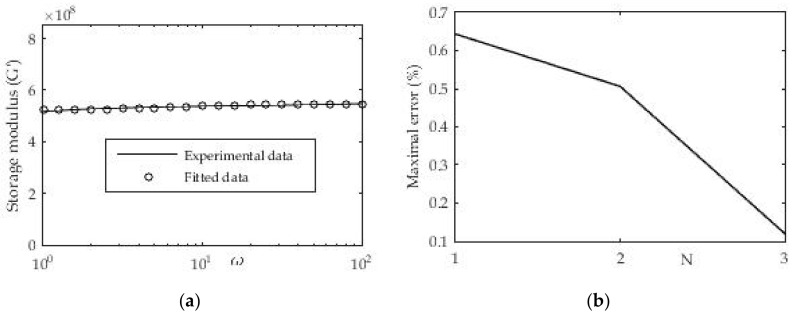
(**a**) Approximation of shear modulus with three terms in Prony series and one free member at temperature T = 20 °C; (**b**) Maximal deviation ratio of experimental and fitted data.

During this dynamic test, the frequency changes, while the amplitude of deformation or the amplitude of the shear stress is kept constant. Data at low frequencies describe the behavior of samples at slow load changes. Analogous to this, the fast load behavior is expressed at high frequencies. For this example, ABS material was taken from which test samples were made and tested on a rheometer. The relaxation module was presented in the form of a Prony series where the number of members gradually varied towards higher values. The results are presented in the following Figure 12 and Table 5.

To approximate the mentioned experimental data, expression (8) with the previously mentioned nonlinear regression was used. From the presented Figure 12 we have a good agreement of experimental and approximated results. The results can also be presented in the following tabular form:

For this procedure, the maximum deviation of the measured and approximated value of the shear stress storage module was calculated. Figure 12b shows the decrease in the maximum deviation of the two compared sizes with the increase in the number of members in the Prony series as expected. It can also be seen that in the first approximation with the Prony series in which we have only one member that the deviation is less than 1%, which is quite acceptable.

#### 3.1.5. Determination of Time-Temperature Coefficient a_T_

**Figure 13:**
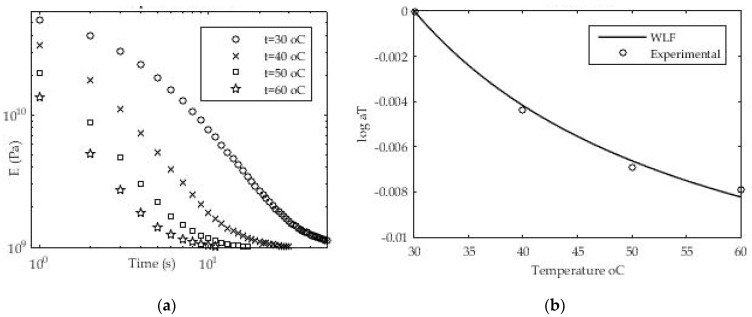
(**a**) Experimental data for relaxation modulus at various temperatures; (**b**) Values for time—temperature shift factor a_T_.

To calculate the shift factor, a series of measurements of the relaxation module at different temperatures starting from T_1_ = 30 °C to T_4_ = 60 °C with an increase of ΔT = 10 °C was performed (Figure 13). The value of the so-called experimental window EW = 5.0 × 10^+1^ s. The above expression (18) was used primarily to determine the shift factor for an individual temperature difference (e.g., 30 °C and 40 °C).

To determine the value of the shift factor between the two isothermal values, a nonlinear regression implemented in MATLAB software was used again, which was run by the “lsqfit” command. The procedure was further repeated for other values of the shift factor between the values of the relaxation module for the reference temperature (in this case T_ref_ = 30 °C) and the values of the relaxation module for the experimental isothermal conditions (T_2_ = 40 °C, T_3_ = 50 °C and T_4_ = 60 °C). After determining the individual values of the shift factor for all temperature values in relation to the reference, expression (19) and nonlinear regression were used to determine the unknown constants C_1_ and C_2_. Using the mentioned methodology and experimental data, the values of WLF constants C_1_ = 0.01593 and C_2_ = 28.13 were obtained for the reference temperature T_ref_ = 30 °C. The values for the time—temperature factor are presented graphically in Figure 13b. A good agreement between the experimental (measured) results and the results obtained using nonlinear regression was found. The MATLAB software package and the “lsqfit” command were used to approximate the experimental results.

### 3.2. Examples of Numerical Simulations

Numerical calculations were performed for polymeric materials for which the relaxation modulus is already known as well as for the material for which the relaxation modulus was determined in a previous experimental analysis. Calculations were performed for pure mechanical load, temperature influence as well as load with simultaneous cooling of the material. Experimental data or available analytical solutions were used to compare the results.

#### 3.2.1. Relaxation Test

**Figure 14:**
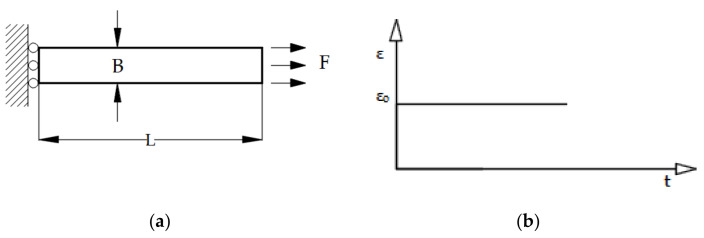
(**a**) Geometry and boundary conditions for the relaxation test (L = 80 mm, B = 10 mm); (**b**) constant loading for the relaxation test.

In this example, the relaxation test for the sample in Figure 14 made of a viscoelastic material is considered. The horizontal movement of the left side of the rod is limited, while the uniaxial load is set on the right side of the rod. The resolution domain is divided into 10 CV with a time step δt = 1 s.

The relaxation modulus of the material in the form of the Prony series was previously experimentally determined and is presented in Table 3. The loads were performed on the right side of the rod by specifying the displacement increment (Figure 14) which can be described in mathematical form as follows:

(30) $$\varepsilon_{o}=5.75\cdot 10^{-2};\;t\geq 0\}\;.\;$$

**Figure 15:**
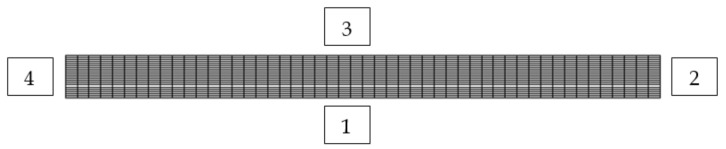
Numerical mesh for 2D domain (50 × 20 CV).

In the given example, 2D spatial discretization with a uniform orthogonal numerical mesh was used, although the example can also be solved in the 1D domain. For regions 2 and 4 the displacement vector is known at the boundary (Equation (22)), for region 1 (Figure 15) the boundary condition is the symmetry plan (Equation (23)), while for region 3 the known (free surface) load is valid, i.e., the boundary condition is also from Equation (22).

**Figure 16:**
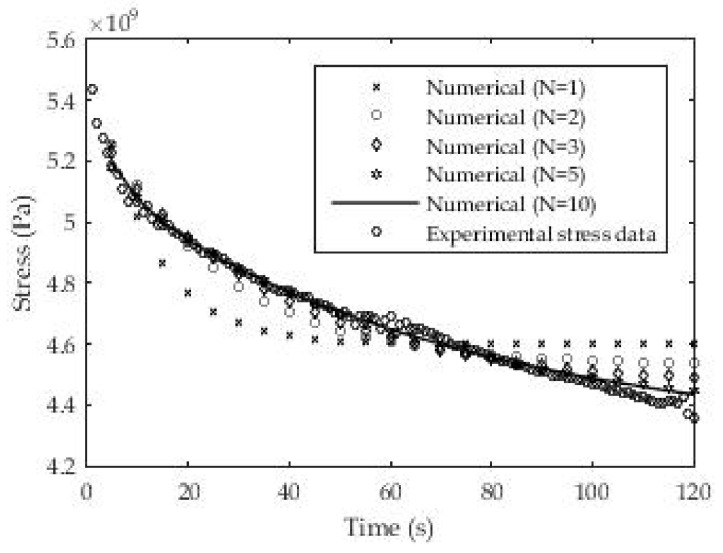
Numerical results for the relaxation test depending on the number members in the Prony series.

To solve this case, a grid with (50 × 20) 1000 control volumes was used. Figure 16 shows that as the number of exponential members in the relaxation module increases, the numerical results increasingly agree with the experimental results.

#### 3.2.2. Creep Recovery Test

**Figure 17:**
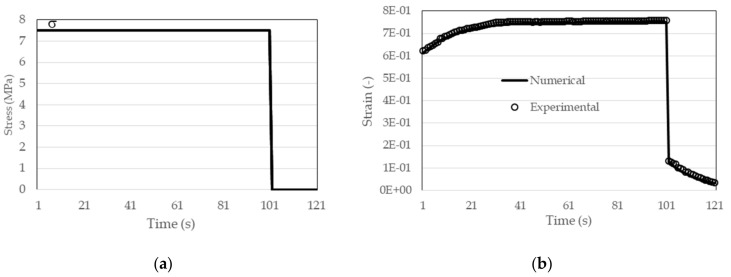
(**a**) Creep recovery test; (**b**) results of creep recovery test.

For the next test, the same material (ABS) was selected from the relaxation test (3.2.1) with identical characteristics of the relaxation modulus, while the load consists of one period of constant load (σ_o_ = 7.5 MPa) to the moment t = 100 s, followed by unloading (σ_o_ = 0 MPa) (Figure 17). The geometry and domain of the solution are identical to those in Section 3.2.1, and the load limit condition was applied to region 2 of Figure 15. Other boundary conditions as well as geometry are the same as in Figure 14a. An expression with one free and one exponential term according to Table 4 was used for the relaxation module.

For this case, an analytical solution can be found that takes the form:

(31) $$\varepsilon \left(t\right)=\sigma_{o}\left[J\left(t\right)-J\left(t-\tau \right)\right]\;,\;$$

where the coefficients in the creep modulus are determined on the basis of a three-parameter model:

(32) $$J_{o}=\frac{1}{E_{o}+E_{1}};J_{1}=\frac{1}{E_{o}}-\frac{1}{E_{o}+E_{1}};r=\tau \left(\frac{E_{o}+E_{1}}{E_{o}}\right)\;,$$

where r is the relaxation time in the creep modulus. More about performing an analytical solution can be found in the literature [11].

In Figure 17b a fairly good agreement of the numerical results with the measured experimental results (−o) is shown. In the same figure it can be seen that the creep phenomenon is most pronounced at the beginning of the test.

#### 3.2.3. Plane-Strain Time-Varying Test

**Figure 18:**
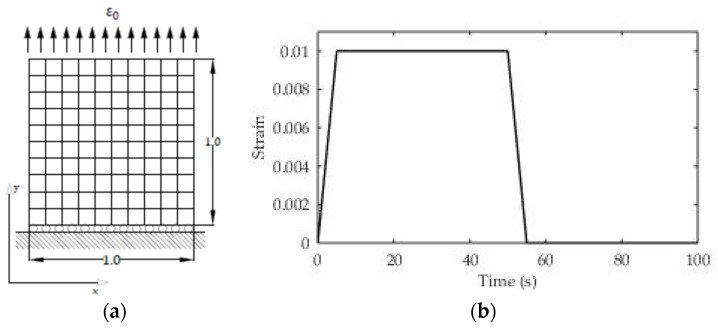
(**a**) Geometry and numerical mesh; (**b**) results of creep recovery test.

In this case, the material with the experimentally determined relaxation modulus from Table 3 was also used. The relaxation module is characterized as a Prony series with two exponential terms and one free term. The sample density is known and is 900 kg/m^3^. Poisson’s ratio for ABS is taken from the literature (υ = 0.35). The geometry and boundary conditions are given in the following Figure 18a. This test case is analyzed in the 2D domain, and since no analytical solution is known, the obtained simulation results are compared with the results of a numerical simulation carried out using the finite element method. The load at the boundary of the solution domain consists of a time-varying strain, while the total test time is divided into four regions.

The first region consists of a linear increase in deformation to a value of 1% (ε_o_ = 0.01, t_1_ = 5 s), the second is the maintenance of constant deformation until t_2_ = 50 s, in the third region a linear unloading is performed until t_3_ = 55 s (ε_o_ = 0), and in the last region the relaxation effect is monitored until t_4_ = 100 s. The time course of the load is also presented in Figure 18b.

**Figure 19:**
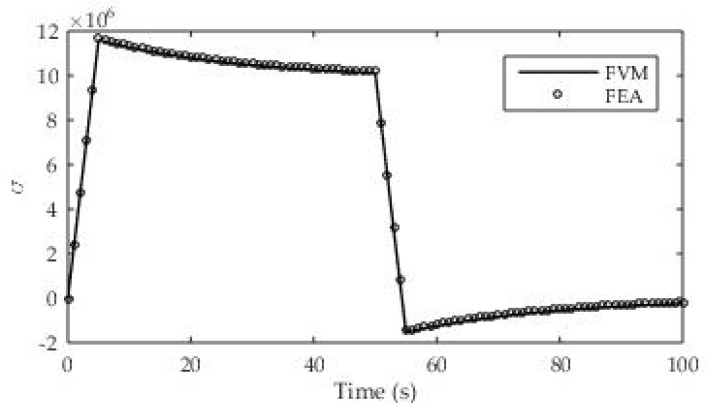
Numerical results for stress relaxation test.

An orthogonal numerical grid of 100 CVs was used to discretize the solution domain (Figure 18a), while a step of t = 1 s was chosen for time discretization. Figure 19 presents the numerical results obtained using two numerical methods (Finite Volume Method and Finite Element Analysis). Two numerical methods were used due to the very difficult performance of an experiment of this type, and an analytical solution for this test case (υ ≠ 0) is not available. The application of the numerical finite element method was performed using the well-known ABAQUS software with built-in subroutines for calculating viscoelastic deformations and stresses. The numerical solutions agree very well in all load regions and good agreement is especially visible at linear loading or unloading.

#### 3.2.4. Analysis of Viscoelastic Material with Simultaneous Mechanical and Thermal Loading

For this case, the material that was previously subjected to experimental analysis in determining the relaxation modulus in Section 3.1.3 was analyzed. This is a very convenient test example because the numerically obtained data can be further directly compared with the experimental data. The thermal effect is taken into account through the use of shift factors in the form of a WLF function. The values for the coefficients in the WLF function were taken from Section 3.1.4 since the same material was used. Other physical characteristics are presented in Table 6, below.

**Figure 20:**
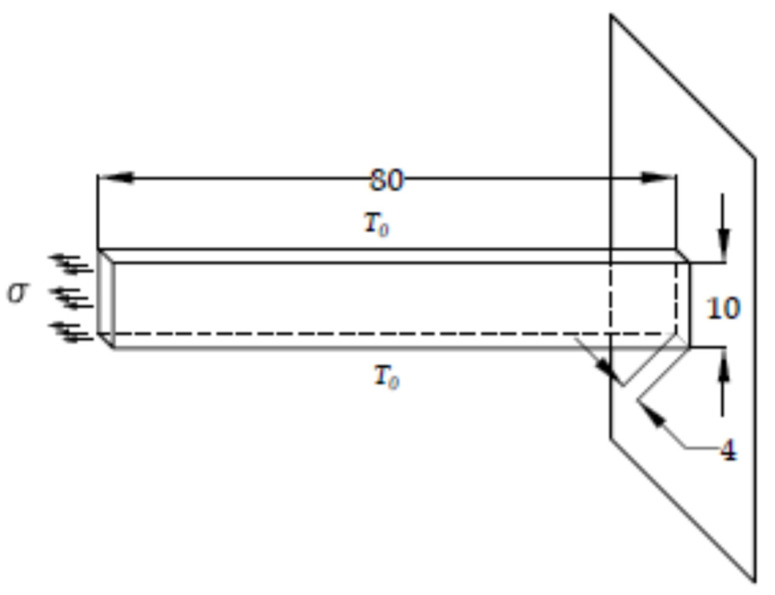
Geometry and boundary conditions.

Geometrical characteristics and boundary conditions are presented in Figure 20 where the initial temperature T_1_ = 30 °C at all points of the domain.

The material is gradually heated by means of a linearly changed temperature T_o_. The design of the test device is such that a sample is fixed at one end in the experimental chamber while a constant stress load is set at the other end. The temperature rate is constant and after t = 180 s it is 60 °C. The load at the end of the rod is σ = 1.51× 10^+5^ Pa.

**Figure 21:**
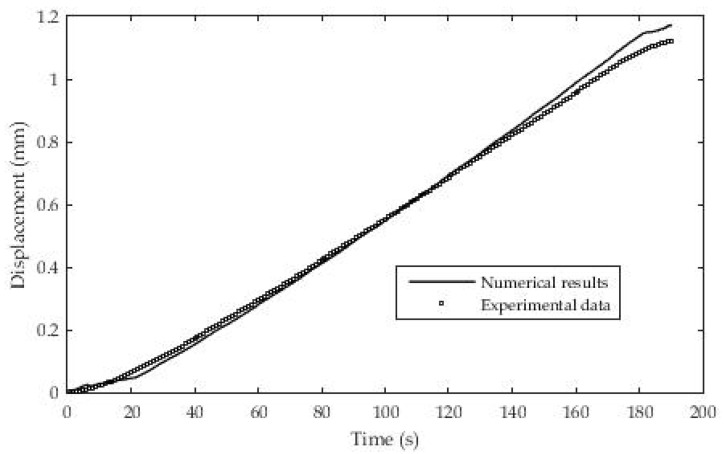
Experimental and numerical data for displacement.

After the geometry was made and the initial and boundary conditions were applied, the following results were obtained as in Figure 21. The presentation of the results shows a fairly good agreement between the results obtained using the numerical method in relation to the experimentally determined results from moving the end of the rod.

## 4. Discussion

The experimental research of relaxation functions presented in this paper can be applied to a wide range of polymeric materials, starting from “pure” plastics, such as ABS, to materials formed as a mixture of polymers and some basic material (e.g., wood or a combination of wood and glue). The selected application examples demonstrate the specific determination of the coefficients in the Prony series based on the experimental values of the relaxation function. Two examples of determining the analytical form of the relaxation function show that the methodology for determining the unknown coefficients in the Prony series is applicable in the real time (t) domain as well as in the angular velocity (ω)-domain. After determining the analytical expression under isothermal conditions, the influence of temperature on the values of the relaxation function was investigated, where the time-temperature principle was applied. The application of this principle was performed within the experimental window, where the “shift-factor” values were determined using the WLF equation and nonlinear regression between the experimental values and the assumed analytical expression. After this analysis, a numerical calculation was performed for non-isothermal conditions with such an experimentally determined relaxation function and shift factor, with a small difference between the numerical and experimental results. A rheometer test device was used for testing the samples, as well as a universal ZwickRoell compression and extension testing machine. Both devices provided quite good control of the input parameters, primarily the control of the sample load, whether it was a creep or relaxation test. Nonlinear regression, i.e., the well-known least squares method using the subroutines in MATLAB, very quickly gave good results of “fitting” the experimental data for an arbitrary and optimized set of coefficients in the Prony series. For the application of the recursive algorithm, the finite volume method was used, which was implemented in earlier papers for the problems of viscoelastic solids. The basis for all numerical calculations is the software package SA-2D developed for the analysis of thermo-viscoelastic deformations. Finally, it was shown that for polymer 3D printed materials a relaxation function can be determined in the form of a Prony series based on the experiments performed in this study. Based on the analytical expressions of the Prony series and shift factors, the numerical calculations yielded results that differed very little from the experimental ones. The numerical results using the recursive algorithm did not deviate significantly from the experimental ones even when considering problems with non-isothermal conditions.

## Figures and Tables

**Table 1:** Coefficients in creep modulus (PLA, d_1_ = 0.09 mm).

	Number of Terms		Number of Terms		Number of Terms		Number of Terms	
	1		3		5		7	
i	J_i_	τ_i_	J_i_	τ_i_	J_i_	τ_i_	J_i_	τ_i_
0	3.3 × 10^−9^		3.2 × 10^−9^		3.1 × 10^−9^		2.9 × 10^−9^	
1	6.7 × 10^−10^	1.0 × 10^3^	1.6 × 10^−10^	1.0 × 10^1^	2.3 × 10^−11^	1.0 × 10^−1^	3.8 × 10^−11^	1.0 × 10^−3^
2			1.3 × 10^−12^	1.0 × 10^2^	3.3 × 10^−14^	1.0 × 10^−0^	3.0 × 10^−10^	1.0 × 10^−2^
3			6.5 × 10^−10^	1.0 × 10^3^	1.5 × 10^−10^	1.0 × 10^1^	5.0 × 10^−11^	1.0 × 10^−1^
4					8.7 × 10^−12^	1.0 × 10^2^	4.0 × 10^−11^	1.0 × 10^−0^
5					6.4 × 10^−10^	1.0 × 10^3^	1.3 × 10^−11^	1.0 × 10^1^
6							3.2 × 10^−11^	1.0 × 10^2^
7							9.2 × 10^−10^	1.0 × 10^3^

**Table 2:** Average deviation between experimental and fitted data (PLA, d_1_ = 0.09 mm).

	Number of Terms	Number of Terms	Number of Terms	Number of Terms
	1	3	5	7
Average error (%)	1.8	1.6	0.9	0.8

**Table 3:** Prony series for ABS material and layer printing d = 0.19 mm.

	Number of Terms		Number of Terms		Number of Terms		Number of Terms	
	1		2		5		10	
	τ_i_ (s)	J_i_	τ_i_ (s)	J_i_	τ_i_ (s)	J_i_	τ_i_ (s)	J_i_
0		1.00 × 10^+9^		9.85 × 10^+8^		9.46 × 10^+8^		9.25× 10^+8^
1	10.95	2.25 × 10^+8^	4.93	3.98 × 10^−1^	2.22	7.82 × 10^+7^	1.55	1.28 × 10^+7^
2			24.33	1.94 × 10^+8^	4.93	2.53 × 10^−1^	2.39	1.21 × 10^+7^
3					10.95	4.92 × 10^−1^	3.69	1.89 × 10^+7^
4					24.33	1.96 × 10^−1^	5.70	2.57 × 10^+7^
5					54.03	1.89 × 10^+8^	8.81	2.92 × 10^+4^
6							13.62	4.90 × 10^+6^
7							21.04	1.20 × 10^+7^
8							32.52	2.00 × 10^+6^
9							50.25	1.44 × 10^+6^
10							77.65	1.82 × 10^+8^

**Table 4:** Prony series for ABS material and layer printing d = 0.19 mm.

	Number of Terms		Number of Terms		Number of Terms		Number of Terms	
	1		2		5		10	
	τ_i_ (s)	E_i_ (Pa)	τ_i_ (s)	E_i_ (Pa)	τ_i_ (s)	E_i_ (Pa)	τ_i_ (s)	E_i_ (Pa)
0		1.00 × 10^+9^		9.85 × 10^+8^		9.46 × 10^+8^		9.25× 10^+8^
1	10.95	2.25 × 10^+8^	4.93	3.98 × 10^−1^	2.22	7.82 × 10^+7^	1.55	1.28 × 10^+7^
2			24.33	1.94 × 10^+8^	4.93	2.53 × 10^−1^	2.39	1.21 × 10^+7^
3					10.95	4.92 × 10^−1^	3.69	1.89 × 10^+7^
4					24.33	1.96 × 10^−1^	5.70	2.57 × 10^+7^
5					54.03	1.89 × 10^+8^	8.81	2.92 × 10^+4^
6							13.62	4.90 × 10^+6^
7							21.04	1.20 × 10^+7^
8							32.52	2.00 × 10^+6^
9							50.25	1.44 × 10^+6^
10							77.65	1.82 × 10^+8^

**Table 5:** Prony series for material ABS at temperature T = 20 °C.

Coefficients of Prony Series						
	1 Term		2 Terms		3 Terms	
i	*G_i_*	*τ_i_*	*G_i_*	*τ_i_*	*G_i_*	*τ_i_*
**0**	5.6 × 10^+8^		5.6 × 10^+8^		5.7 × 10^+8^	
**1**	1.5 × 10^+7^	1.0 × 10^−1^	1.1 × 10^+7^	3.3 × 10^−1^	1.5 × 10^+9^	2.4 × 10^−5^
**2**			1.0 × 10^+7^	3.1 × 10^−2^	1.5 × 10^+7^	6.4 × 10^−2^
**3**					5.7 × 10^+5^	3.2 × 10^+2^

**Table 6:** Physical properties of ABS—plastics and layer d = 0.19 mm.

Density ρ (kg/m^3^)	Heat Conductivity λ_t_ (W/m^2^K)	Linear Thermal Expansion α (K^−1^)
900	0.1	2.2× 10^−4^

## Nomenclature

a, b	coefficients in discretized equations
a_T_	shift factor
c	specific heat
d	location vector
e	internal energy
E	Young’s relaxation modulus
f	force
G	shear relaxation modulus
I	unit tensor
i	Cartesian unit vector
J	creep compliance
K	volume relaxation modulus
k	thermal conductivity
P_0_, P_j_	centers of control volumes
q	heat flux vector
Q, q	source terms
r	distance vector
n	vector of outer normal
t	time
t’	reduced time
T	temperature
U	total displacement vector
u	displacement vector
V	volume
α	thermal expansion coefficient
Ґ	diffused term
δt	time increment
δT	temperature increment
δε	strain increment
λ, μ	Lame’s coefficients
ρ	density
σ	stress tensor
τ	incremental time
ν	Poisson’s number
ω	angular velocity
φ	generic variable
γ	angular strain
Subscripts	
B	boundary
i	Cartesian components
j	cell index (volume)
k	index in the Prony series
r	radial direction
x,y,z	Cartesian coordinates
Superscripts	
i	time-step counter
j	iteration counter
T	transpose
Abbreviations	
DMA	dynamic-mechanic analysis
FDM	fused deposition modeling
PLA	poly-lactic acid
ABS	acrylonitrile butadiene styrene
PE	polietilen
CFS	closed-form shifting
TTSP	time temperature
	superposition principle
FE	finite element method
FVM	finite volume method
